# QTL detection and genomic prediction for resistance to anthracnose in alfalfa (*Medicago sativa*)

**DOI:** 10.1002/tpg2.70085

**Published:** 2025-08-06

**Authors:** Marie Pégard, Camille Gréard, Marius Grelier, Marie‐Christine Gras, Laure Saint‐Pierre, Bernard Tharel, Philippe Barre, Bernadette Julier

**Affiliations:** ^1^ INRAE, P3F Lusignan France; ^2^ GIE GRASS Saint‐Sauvant France; ^3^ Cérience Saint‐Sauvant France; ^4^ RAGT 2n Druelle France; ^5^ Barenbrug Connantre France

## Abstract

Forage production, persistence, and associated ecosystem services provided by the major forage legume, alfalfa (*Medicago sativa*), may be affected by disease susceptibility. Resistance to anthracnose, caused by *Colletotrichum trifolii*, has been described as an oligogenic trait, but the precise location of resistance genes on the alfalfa genome is not known. Therefore, we phenotyped a set of 417 alfalfa accessions for anthracnose resistance as the frequency of resistant plants. With available genotyping by sequencing data for 380 accessions from this collection, we performed quantitative trait locus (QTL) detection by genome‐wide association study (GWAS) and genomic prediction using a validation set of 97 accessions randomly selected. A wide range of variation for anthracnose resistance was observed, with newer varieties and breeding materials exhibiting greater resistance than old varieties and landraces. Accessions from America showed the highest resistance, although some European accessions also displayed notable resistance. Six QTLs, controlling 58% of the variation, were identified by GWAS. Two major QTLs were found on chromosome 8, within a region already identified in an alfalfa mapping population. Four other QTLs, each controlling less than 5% of the variation, were also found, including one near a major QTL on chromosome 4 in the model species *M. truncatula*. The predictive ability of our set of accessions was surprisingly high: 85%. These results are promising and highlight the potential of molecular markers and genomic prediction to improve anthracnose resistance in alfalfa breeding programs.

## Core Ideas

- We report a large diversity for anthracnose resistance among alfalfa accessions.
- Two large quantitative trait loci were identified by genome‐wide association study on chromosome 8 within a short distance.
- The genomic prediction of anthracnose resistance reached 0.85.
- Marker‐assisted breeding could improve anthracnose resistance.

**Abbreviations**
GBLUP: genomic best linear unbiased prediction
GBS: genotyping by sequencing
GWAS: genome‐wide association study
MLMM: multi‐locus mixed model
PDA: potato dextrose agar
QTL: quantitative trait locus

## 1 INTRODUCTION

Alfalfa or lucerne (*Medicago sativa* L.) is a forage crop of great interest, especially in the context of the increasing global demand for locally produced protein (Dubeux et al., 2024; Julier et al., 2017; Singer et al., 2018). This species is an autotetraploid with a genome size of 2738 Mb (Chen et al., 2020). This complexity makes breeding and marker‐assisted selection more difficult and slower. Moreover, it also provides numerous other ecosystem services such as breaking pests and diseases’ cycles, improving soil structure, and saving fossil energy. However, Southern anthracnose, caused by *Colletotrichum trifolii*, is one of the most important diseases of perennial alfalfa worldwide, responsible for significant yield losses (Nutter et al., 2002; Yang et al., 2007). This pathogen causes dry necrosis on the stems and the leaves, resulting in defoliation and yield losses of up to 25%–30%, as well as a vigor loss in the spring following the infection (Barnes et al., 1969; Irwin et al., 1980).

**FIGURE 1:**
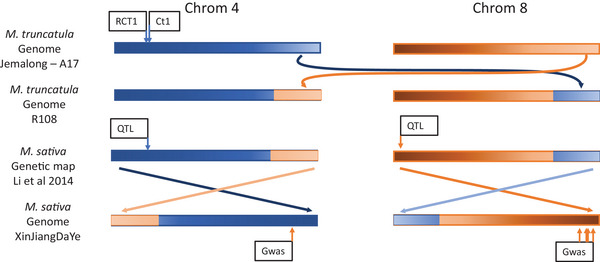
Synthetic view of alignment among *M. truncatula* genomes of Jemalong‐A17 v5 (Pécrix et al., 2018) and R108 (A. Li et al., 2022), *M. sativa* genetic map (X. H. Li et al., 2014), and genome XinJiangDaYe (Chen et al., 2020) for chromosomes 4 and 8. The translocation between the bottom of chromosomes 4 and 8 of Jemalong‐A17 and R108, conserved in *M. sativa* is represented. The arrows represent the genes or quantitative trait loci (QTLs) found in previous studies: *RCT1* (Yang et al., 2007), *Ct1* (Ameline‐Torregrossa et al., 2008a), two QTL in a mapping population (Mackie et al., 2007). The boxes Gwas indicate the single‐nucleotide polymorphism (SNP) found with genome‐wide association study (GWAS) in this study.

Breeding for resistance to anthracnose mainly uses two pathotypes (described as “races” in the literature) of *C. trifolii*, defined by the resistance level of two American reference varieties: Arc, resistant to pathotype 1 and susceptible to pathotype 2, and Saranac AR, resistant to both pathotypes (Ostazeski & Elgin, 1982). An inheritance study showed that the resistance of Arc to pathotype 1 was controlled by the dominant allele of the *An_1_* gene, whereas the resistance of Saranac AR to pathotypes 1 and 2 was controlled by another independent gene *An_2_* (Elgin and Ostazeski, 1985). In Europe and North Africa, different isolates were collected in alfalfa fields showing anthracnose symptoms, and the varieties showing resistance to pathotype 1 were also resistant to these isolates (Gondran, 1982; Gosset et al., 1989; Raynal, 1977; Raynal et al., 1989). Resistance to the pathotype 1 and the French isolates was also described to be only partially dominant (Guy, 1976; Mackie et al., 2007). Studies of the genetics of resistance to anthracnose pathotype 1 in the model species *Medicago truncatula* reported a major locus of resistance in the upper part of chromosome 4, named *RCT1* (Yang et al., 2007) or *Ct1* (Ameline‐Torregrosa, Cazaux, et al., 2008). The resistant allele of the *RCT1* gene, identified as a member of the Toll‐interleukin‐1 receptor/nucleotide‐binding site/leucine‐rich repeat (TIR‐NBS‐LRR) class of plant R genes, conferred anthracnose resistance when transferred into susceptible alfalfa plants (Yang et al., 2008). In an alfalfa mapping population that was a backcross between a resistant clone and a susceptible one, six quantitative trait loci (QTLs) for resistance to pathotype 1 were identified, including a major QTL at the top of chromosome 8 and a minor QTL at the top of chromosome 4, on a homeologous region of *M. truncatula RCT1* (Mackie et al., 2007). The positions of the QTL identified so far on alfalfa and *M. truncatula* genomes have been synthetized and aligned with the first alfalfa reference genome (Chen et al., 2020) (Figure 1). Before the release of this alfalfa genome sequence, as *M. truncatula* was the model species for legume crops, most of the *M. sativa* genetic maps and genomes were aligned on the successive versions of the *M. truncatula* reference genome of Jemalong‐A17 (Pecrix et al., 2018; Young et al., 2011). This first alfalfa reference genome was compared to an *M. sativa* genetic map (Chen et al., 2020) that was itself aligned to the original reference genome of *M. truncatula* (X. H. Li et al., 2014). This evidenced that the reference genome was not orientated as the *M. truncatula* genome, and namely, chromosomes 1, 3, 5, and 6 were in the same orientation, while chromosomes 2, 4, 7, and 8 were inversed. In addition, there was a translocation between the bottom of chromosome 4 and the bottom of chromosome 8 when comparing between *M. truncatula* Jemalong‐A17 genome and other *M. truncatula* genotypes (A. Li et al., 2022), and this translocation was conserved in *M. sativa* (Chen et al., 2020; X. H. Li et al., 2014). The schematic view of the genome alignment is summarized in Figure 1.

Selection for anthracnose resistance in breeding programs is a key lever for controlling anthracnose in alfalfa (Elgin et al., 1981; Mackie et al., 2007). Recurrent selection is a method to obtain varieties with a certain level of resistance to *C. trifolii* (Clements et al., 1984; Devine et al., 1971). At the individual level, each plant is scored as susceptible or resistant. At the variety level, because of their synthetic structure, the resistance is expressed as the proportion of resistant plants. Due to the variety heterogeneity, the high level of heterozygosity (Flajoulot et al., 2005; Valizadeh et al., 2011), the severe inbreeding depression (Busbice & Wilsie, 1966; Wilsie, 1958) of this autotetraploid species (2*n* = 4*x* = 32), and the dominance of the resistance, a certain proportion of susceptible plants is maintained in the varieties considered as resistant (Mackie et al., 2007). So far, markers found to be associated with anthracnose resistance have not been used in breeding programs. Indeed, the loci identified in *M. truncatula* have not been proven to be responsible for resistance in alfalfa, and because of the short linkage disequilibrium in alfalfa (Herrmann et al., 2010), the QTL found in an alfalfa mapping population (Mackie et al., 2007) cannot be used in other populations.

The recent release of alfalfa reference genome sequences (Chen et al., 2020; W. Y. Li et al., 2024; Long et al., 2022; Shen et al., 2020; Shi et al., 2024) is key information to detect QTL with genome‐wide association study (GWAS) and to build genomic prediction equations (Flint‐Garcia et al., 2003; Meuwissen et al., 2001). These methods have already been applied to several agronomic traits (Annicchiarico et al., 2015, 2021, 2022; X. Jiang et al., 2024; X.‐Q. Jiang et al., 2022; Murad Leite Andrade et al., 2022; Pégard et al., 2023), with a prediction ability ranging from 0.2 to 0.8 depending on the population or the method. Previous studies have explored various statistical models for genomic prediction in alfalfa, focusing on improving prediction accuracy for agronomic traits. Traditional methods such as genomic best linear unbiased prediction (GBLUP) and Bayesian approaches (e.g., BayesA, BayesB, BayesC) are commonly used as baseline models and often perform similarly. Some studies have shown that machine learning methods, particularly support vector machine (SVM), often outperform traditional statistical models in terms of prediction accuracy. For instance, Zhang et al. (2023) reported that SVM regression achieved the highest prediction accuracy (64.1%) for autumn dormancy, surpassing other models such as Lasso and ElasticNet. However, with the same population, we found that Lasso and GBLUP performed similarly for autumn dormancy, even when major QTLs were detected (Pégard et al., 2023). To our knowledge, genomic prediction has not been used in relation to anthracnose resistance. GWAS and genomic prediction could help to decipher the genetic control of anthracnose resistance in alfalfa and to detect trait‐associated markers that could be used in breeding programs. The selection of anthracnose‐resistant alfalfa could be accelerated by using such markers.

**Core Ideas**

- We report a large diversity for anthracnose resistance among alfalfa accessions.
- Two large quantitative trait loci were identified by genome‐wide association study on chromosome 8 within a short distance.
- The genomic prediction of anthracnose resistance reached 0.85.
- Marker‐assisted breeding could improve anthracnose resistance.

This study reports on the testing of resistance to anthracnose in a large set of alfalfa accessions that have been genotyped with genotyping‐by‐sequencing (GBS) markers in a previous work (Pégard et al., 2023). Resistance variation was analyzed in relation to geographical origin and year of registration. GWAS was performed, and the ability to predict anthracnose resistance with genomic prediction was assessed. Finally, the potential use of the results for marker‐assisted selection and genomic selection was discussed.

## 2 MATERIALS AND METHODS

### 2.1 Materials

A total of 400 alfalfa accessions were collected worldwide to cover the diversity of the cultivated pool within the dormancy range of 3–7, as described by Pégard et al. (2023). In addition, 17 breeding materials and three varieties were added. In this population of 417 accessions (Table S1), there is an over‐representation of European varieties with both recent materials, including breeding materials, and old varieties or landraces. Accessions from the United States were mostly recent varieties or breeding materials. For all varieties, the country of origin or registration was noted. The status of the accessions (code 300 for landraces, 400 for breeding materials, and 500 for registered varieties) was recorded (https://www.genesys‐pgr.org/documentation/basics#mcpd‐sampstat). The set included 23 landraces, 86 breeding materials, and 308 registered varieties. For 267 varieties, their registration year was available. For the landraces, the year 1950 was attributed as the registration year.

### 2.2 Genotyping

The procedures for DNA extraction, optimization of the GBS methodology, and GBS have been previously documented in Julier et al. (2018). In summary, each accession consisted of 100 plants, and DNA was extracted from a pooled sample of 100 leaflets, with each leaflet taken from an individual plant. This method has been demonstrated to effectively estimate the allele frequency within an accession (Julier et al., 2018). For the double‐digest GBS on alfalfa, the enzymes PstI–MseI were employed to achieve a sufficient number of loci while reducing missing data and accounting for the read count per accession. The reads were mapped to the reference genome of the Chinese variety XinJiangDaYe (Chen et al., 2020), using haplotype 2 of each chromosome. After a series of trimming steps (Pégard et al., 2023), 248,060 markers with less than 5% missing data per position were obtained. The genomic relationship matrix (**G**) was calculated with the subset of 118,418 single‐nucleotide polymorphisms (SNPs) without missing values (Pégard et al., 2023). Because of poor sequencing quality for some accessions combined with seed shortage, genomic information was available for 380 populations in this study.

### 2.3 Inoculum production and inoculation

The *C. trifolii* strain used in this study was the C86‐2. This strain was isolated in 1972 in Lusignan (France) and has been shown to be similar to pathotype 1 (Gondran, 1982; Gondran & Mainer‐Casado, 1973). *C. trifolii* was cultivated on a potato dextrose agar (PDA) medium and conserved at −80°C. For spore multiplication, a drop of defrost spore solution was first spread on a sterile PDA medium (10 mL of inclined medium in a 2.2‐cm diameter tube). The tube was then exposed to natural light or natural‐sunlight‐imitating light at 20°C. *C. trifolii* sporulated after 1 week, when a pink jelly‐like substance could be observed at the surface of the black mycelium. The jelly was scratched with a sterile inoculating loop and introduced into 2 mL of sterile water. After homogenization, 0.5 mL of spore suspension was spread in Roux flasks containing PDA medium horizontally jellified. The flasks were exposed to light at 20°C for 1 week. Once the sporulation occurred, 50 mL of sterile water was poured into each flask, which was then closed and gently shaken to collect the jelly substance containing spores. This spore solution was filtered. Spore concentration was evaluated by counting spores on a Malassez cell and diluted to a concentration of 1–2 × 10^6^ spores/mL for the inoculation.

### 2.4 Phenotyping

Anthracnose resistance is studied as part of variety evaluation tests since 2010. All varieties registered in France from 2010 along with varieties from the reference collection were thus characterized for anthracnose resistance by GEVES. GEVES is the French registration office responsible for variety evaluation and seed testing. Of the 417 accessions, GEVES made the results of 150 accessions available to us. In addition, three private breeders active in France (Barenbrug, Cérience, and RAGT 2n) phenotyped 295 accessions, of which 28 accessions were tested both by the private partners and GEVES (Table 1). Three to four control varieties (Ambra, Marshal, Alexis, and Kali), marketed in Europe and showing a resistance level ranging from highly resistant to highly susceptible, were included in each test as control varieties.

**TABLE 1:** Series of anthracnose tests at GEVES and at three breeders’ places, number of repetitions, and number of accessions, including three to four control varieties, in each test.

Test identifier	Number of repetitions	Number of accessions
GEVES 12_3	3	32
GEVES 13_1	3	38
GEVES 14_1	3	44
GEVES 15_1	3	35
GEVES 15_2	2	29
GEVES 16_1	3	19
GEVES 17_1	3	15
GEVES 18_1	3	20
GEVES 18_2	3	8
GEVES 19_1	3	24
GEVES 20_1	3	18
GEVES 21_1	3	26
Barenbrug 1	3	37
Barenbrug 2	2	14
Barenbrug 3	4	48
Barenbrug 4	4	50
Cérience 1	3	49
Cérience 2	3	50
Cérience 3	3	50
RAGT 2n 1	3	56
RAGT 2n 2	3	41

The protocol used by GEVES and in this study (Perrot et al., 2009) is similar to the protocol used in the United States (O'Neill, 2022). For each accession, the seeds were placed on blotting paper at 25°C under continuous light for 24–72 h for germination. Then, 50–90 germinated seeds per repetition were planted in seedling trays placed in a growth chamber at 25°C/23°C day/night, with a 12‐h photoperiod. The number of repetitions and accessions for each test are indicated in Table 1. Plants were inoculated with *C. trifolii* C86‐2 at the first trifoliate leaf stage. Before inoculation, the number of living plants in each repetition of each accession was counted. Inoculation was performed by evenly spraying 130–160 mL spore suspension with a concentration of 1–2 × 10^6^ spores/mL per tray, as described in Gondran and Mainer‐Casado (1973). Plant trays were then placed back in the growth chamber under the same conditions (25°C/23°C day/night, with a 12‐h photoperiod), with a saturated hygrometry for the first 48 h. Resistance was scored 15 days after inoculation by evaluating the symptoms on each inoculated plant to classify them into two categories—dead plants (susceptible) and non‐dead plants (resistant)—without visible symptoms or with wilting signs.

### 2.5 Data analysis

The percentage of resistance (*y*) was adjusted for trial and repetition effects using the following mixed model:

y=μ+TR+X+ε

where *μ* is the overall mean, TR is the combination of the trial and repetition effects, treated as a random effect, *X* is the genetic effect also considered as a random effect, and *Ɛ* is the residual effect. Notably, the accession identification was used as the genetic effect rather than the kinship between accessions based on markers. This approach was necessary because some of the control varieties were not genotyped. The percentage of resistance was adjusted by removing the component explained by the trial–repetition combination from the raw percentage. Subsequently, the adjusted percentage was used as the phenotype for further analysis. The percentage of resistance was represented as a histogram to illustrate the range of variation. It was plotted as a function of the registration year, when available, and the accession status (landrace, breeding material, registered variety). The mean resistance score standard deviation was calculated for each country of origin. The broad‐sense heritability was estimated using the variances calculated with the above model as follows:

VarX/(VarX+VarTR+Varε).

The genomic relationship matrix **G** was based on VanRaden (2008), adapted to use allele frequencies (continuous values from 0 to 1) instead of allele dose (Ashraf et al., 2014). The genotyping matrix **M** was normalized by the minimum allele frequency (*p*) to obtain the normalized genotyping matrix **Z** used to compute **G** as follows:

$$G=\frac{{\mathrm{ZZ}}^{\prime }}{\frac{1}{n}\sum_{j=1}^{m}p_{j}\left(1-p_{j}\right)}$$

The denominator is a scaling parameter, corresponding to the sum of the expected SNP variance across genotypes (Ashraf et al., 2014), where *m* represents the number of markers, *p_j_* equals the frequency of the *j*th marker, and *n* represents a scaling number to obtain a diagonal mean close to 1. This has been recommended in previous studies on polyploid species (Ashraf et al., 2014; Cericola et al., 2018), and with *n *= 16, the diagonal mean was close to 1.

GWAS was conducted using the multi‐locus mixed model (MLMM) method (Segura et al., 2012), which accounted for the genetic structure of the cultivated material through a genomic relationship matrix. This method employed a stepwise mixed‐model regression approach, incorporating SNPs as cofactors through forward inclusion and backward elimination. At each step, the variance components of the model were re‐estimated, enhancing detection power while reducing the false detection rate. The analysis was limited to a maximum of 10 steps, with the optimal step selected using an adjusted multiple Bonferroni criterion (10^−5^). The percentage of phenotypic variation explained by each QTL was determined by comparing the *R* ^2^ values of a linear model that included all QTLs as fixed effects and the genomic relationship matrix (**G**) as a random effect, with and without the focused QTL.

The genes located under the QTL were determined using the Genome Browser developed on the XinJiangDaYe genotype of *M. sativa* https://bbric‐pipelines.toulouse.inra.fr/myGenomeBrowser?search=1&portalname=MSAT_XinJiangDaYe&owner=sebastien.carrere@inrae.fr&key=PyG9k9tK. The positions of the QTL were compared to other QTLs found on *M. truncatula* when the available reference genome sequence was version 1 or a genetic map of *M. sativa*. On *M. truncatula*, the QTL pointed to *RCT1* gene and its protein sequence ACF19650.1 (Yang et al., 2007) or *Ct1* gene included in the BAC BF519144 (Ameline‐Torregrosa, Cazaux, et al., 2008). On *M. sativa*, a major QTL for resistance to anthracnose pathotype 1 was found at the top of chromosome 8 (Mackie et al., 2007), and a minor QTL was found at the top of chromosome 4, but, as the surrounding markers are Amplified Fragment‐Length Polymorphism (AFLP), it was not possible to find the exact homologous region on the current *M. sativa* reference genome.

As several QTLs were found in a small region of the genome, an estimation of linkage disequilibrium was calculated as in Pégard et al. (2023). For this region, the genomic sequences of the four homologs of chromosome 8 (Chen et al., 2020) were extracted and aligned with the D‐Genies program available at https://dgenies.toulouse.inra.fr/ with the default options (Cabanettes & Klopp, 2018). The Pearson correlation between allele frequencies of the SNP involved in QTL was also calculated.

For genomic prediction, a validation population consisting of 97 randomly selected accessions was formed, ensuring that each genetic group identified by Pégard et al. (2023) was represented proportionally to its size. The remaining accessions were randomly sampled to evaluate the effect of the training population size. Eight different sample sizes were tested, ranging from 20% of the remaining accessions (59 accessions) to 95% (270 accessions). For each sample size, 10 iterations were performed. The quality of the predictions was assessed by calculating the predictive ability, defined as the Pearson correlation between the adjusted phenotypes and the predicted values. The best linear unbiased prediction based on genomic information (GBLUP) (Meuwissen et al., 2001; Whittaker et al., 2000) was employed to predict the genomic estimated breeding values using all SNPs to compute the genomic relationship matrix (**G**). The analysis was conducted using the R package breedR.

## 3 RESULTS

### 3.1 Range of variation for anthracnose resistance in alfalfa

The four control varieties showed a wide range of variation for the adjusted resistance percentage, with Ambra being highly resistant (80.4%) and Marshal being resistant (70.0%), while Alexis was susceptible (26.2%) and Kali highly susceptible (17.5%). The resistance of these control varieties thus adequately covered the range of variation.

**FIGURE 2:**
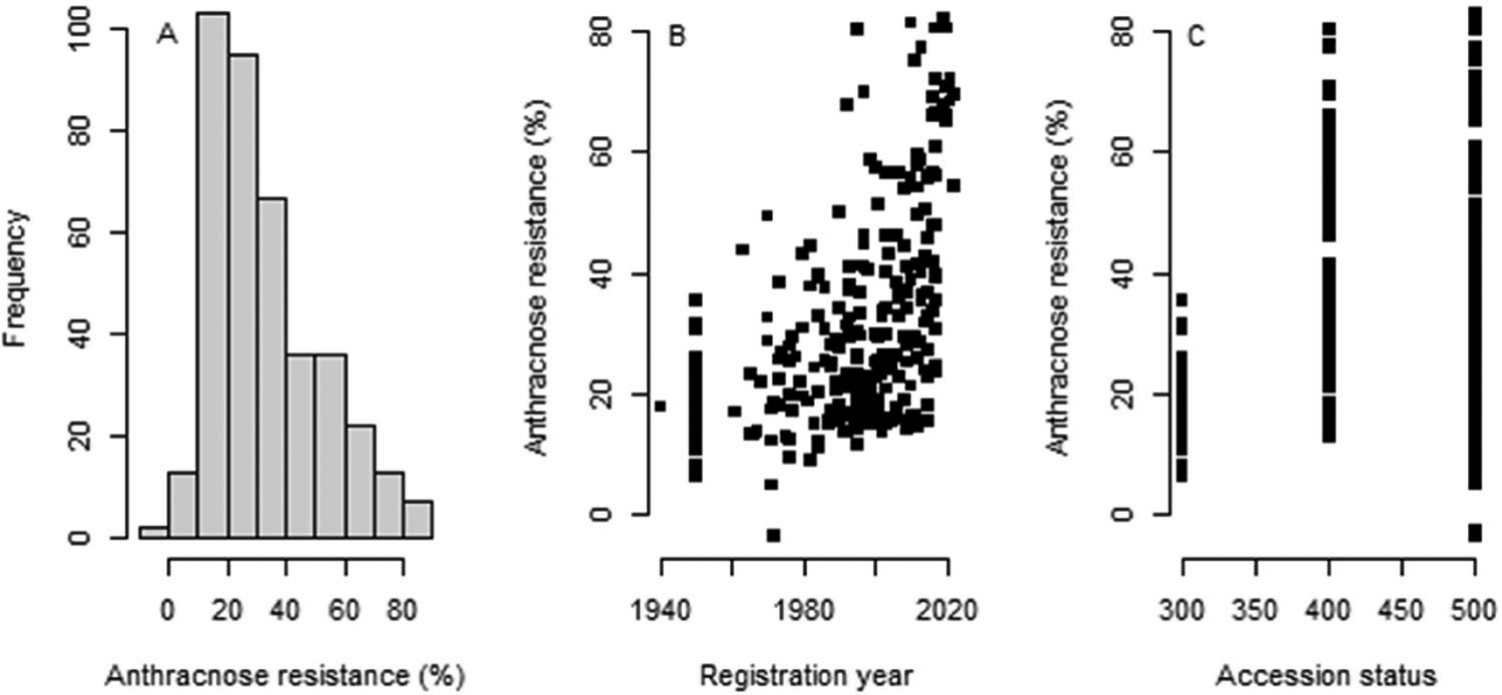
Variation for anthracnose resistance (%) in alfalfa. (A) Range of variation among 417 accessions, (B) resistance of 287 accessions as a function of their registration year, and (C) resistance of 394 accessions as a function of their status (codes 300: landraces, 400: breeding materials, 500: registered varieties).

In the set of accessions, the adjusted resistance percentages ranged from −3.9% to 83.0%, with a mean value of 32.9% (Figure 2A). If we take an arbitrary threshold of 60% resistant plants to define an accession as resistant, then only 59 accessions can be considered resistant. Landraces and old varieties (registered before 1990) were susceptible, with resistances percentages ranging from −3.9% to 49.3% (Figure 2B). After 1990, the varieties had at least resistances above 10%, and most of them had resistances below 40%, but 21 varieties exhibited resistances above 60%. Over the whole set of 417 accessions, 42 accessions (varieties or breeding materials) had resistance percentages above 60%, 26 from the United States, 13 from France, and one from each of China, Italy, and Romania. The landraces were on average susceptible but showed a range of resistance percentages between 6% and 35%, while the breeding materials and the registered varieties covered the full range of variation in resistance percentage (Figure 2C).

**FIGURE 3:**
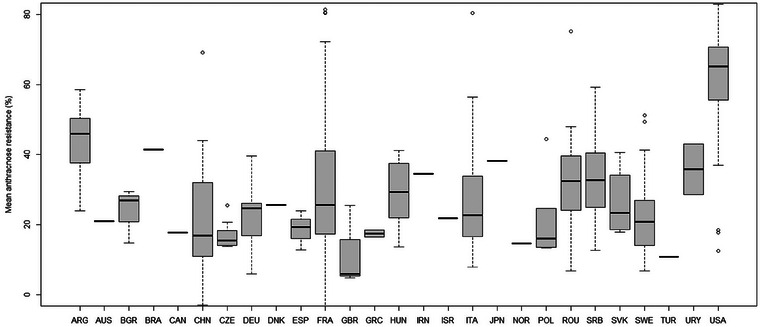
Mean anthracnose resistance (%) in the countries represented in our collection of accessions. When there was more than one accession per country, vertical bars indicate the standard deviation, and dots represent accessions that deviated substantially from the country mean.

The mean resistances were the highest for the accessions from the United States, Argentina, and Uruguay. Accessions from France, Romania, Serbia, Hungary, and Italy generally had intermediate mean resistance. Most of the accessions of the other countries had a resistance below 30% (Figure 3).

### 3.2 QTL detection

The heritability of the resistance percentage was high, estimated at 0.53. Each of the first six steps of MLMM procedures detected a significant QTL (Table 2, Figure S1). Two major QTLs were detected, corresponding to the SNP chr8_80198129 and chr8_83798444, both located at the bottom of chromosome 8 that comprises about 85 Mb (Chen et al., 2020). Among the other four QTLs, two were also located at the bottom of chromosome 8 (chr8_77736763, chr8_80205951), one was at the top of chromosome 2 (chr2_1220775), and the fourth was at the bottom of chromosome 4 (chr4_76542639). The QTL detected in the fifth step, SNP chr8_80205951, was located in the gene just next to the first QTL, SNP chr8_80198129. As the chromosomes of this reference genome were not orientated as in the reference genome of *M. truncatula* (Figure 1), the four QTLs at the bottom of chromosome 8 correspond to the top of chromosome 8 of *M. truncatula* and the QTL at the bottom of chromosome 4 correspond to the top of chromosome 4 of *M. truncatula*. The homeologous region to the *M. truncatula RCT1* gene (Yang et al., 2007) in the *M. sativa* genome spanned from 86665076 to 86671634 on chromosome 4, about 10 million bp from the QTL chr4_76542639. The QTL *Ct1* (Ameline‐Torregrosa, Cazaux, et al., 2008) that corresponded to the BAC BF519144 on *M. truncatula* was homeologous to MS.gene052809, located between 86940546 and 86948333 on chromosome 4, close to *RCT1* homeologous but far from our QTL.

**TABLE 2:** Positions of the quantitative trait locus (QTL) for anthracnose resistance detected in the first six steps of the multi‐locus mixed model (MLMM) procedure, percentage of variation explained, and description of the candidate gene around the position: gene length, protein length, number of exons, location of the mutation and effect on gene and protein sequences, gene annotation, and homeology with *M. truncatula*.

Position	% of explained variation	Candidate gene in *M. sativa*	Gene length (bp)	Protein length (aa)	#Exons	Mutation	Candidate gene annotation	*M. truncatula* homeolog and annotation
chr8_80198129	39.0	MS.gene37046 (strand +)	3663	491	3	Intron	Leucine‐rich repeat domain superfamily	
chr8_83798444	11.3	MS.gene055334 (strand −)	6294	1 095	7	Exon 6, A → G, synonymous	EamA‐like transporter family	MtrunA17_Chr8g0334751 Putative EamA domain‐containing protein
chr8_77736763	3.6	MS.gene36672 (strand +)	36,672	637	15	Intron	Glutamate/leucine/phenylalanine/valine dehydrogenase signature	MtrunA17_Chr8g0341541 Putative glutamate dehydrogenase (NADP(+))
chr2_1220775	1.4	MS.gene83580 (strand +)	1407	468	1	Exon1, T → A, L → M	Leucine‐rich repeat domain superfamily	MtrunA17_Chr2g0333661 Putative leucine‐rich repeat domain superfamily
chr8_80205951	3.1	MS.gene37047 (strand −)	3136	590	4	Exon 1, T → C, synonymous	Leucine‐rich repeat domain superfamily	
chr4_76542639	3.9	At 159 b from MS.gene82171 (strand +)	4641	339	9	3′ region	Unknown	
Total	58.5							

**FIGURE 4:**
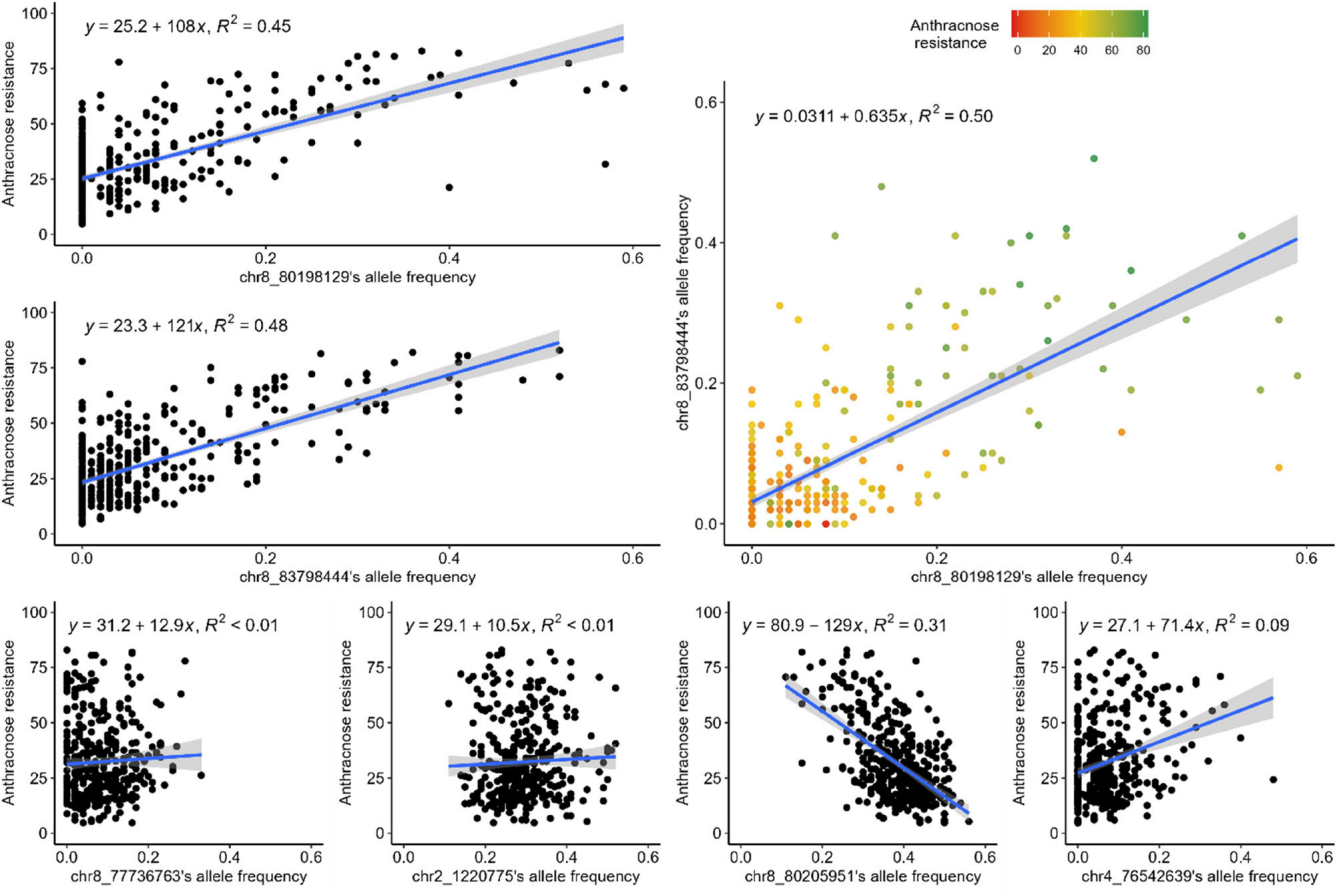
Anthracnose percentage of resistance depending on the allele frequencies of the six quantitative trait loci (QTLs). The two graphs on the top left represent the percentage of resistance (*y*‐axis) depending on the allele frequencies of chr8_80198129 (top) and chr8_83798444 (bottom). The blue line represents the regression between the percentage of resistance of these two QTLs, and the gray part represents the confidence interval of this regression; the equation and *R* ^2^ of that regression are represented on each graph. The graph on the top right represents the allele frequencies of chr8_83798444 (*y*‐axis) depending on the allelic frequencies of chr8_80198129. The points, representing the accessions, are colored depending on their percentage of resistance, from red indicating a low percentage of resistance to green for a high percentage of resistance. The four graphs at the bottom visualize the relationship between anthracnose resistance and the allele frequency of the other four QTLs.

The two major QTLs explained 39.0% and 11.3% of the variation, respectively, while the other QTLs explained only 1.4%–3.9% of the variation (Table 2). Altogether, the six QTLs explained 58.5% of the variation. The frequency of the SNP chr8_80198129 varied from 0 to 0.63 and that of the SNP chr8_83798444 varied from 0 to 0.52 in the set of accessions. For both SNPs, the higher their frequency, the higher the resistance level of the accessions, but no accession had these SNPs at a very high frequency (Figure 4). For the other four QTLs, the correlation between SNP frequency and anthracnose resistance was convincing for the SNPs chr8_80205951 and chr4_76542639, but not for the SNPs chr8_77736763 and chr2_1220775 (Figure 4). For these SNPs as well, the ranges of variation of their allele frequencies did not span from 0 to 1. The two major QTLs were positively correlated (Figure 4, Table 3), the most resistant accessions combining a high frequency for chr8_80198129 and chr8_83798444. Surprisingly, some accessions, such as EUC_MS_254, had high resistance levels but SNP frequencies lower than 0.10 at the two major QTLs, highlighting the role of the second QTL in the resistance. Among all six QTLs, the correlation was significant at *p* < 0.05 between the SNPs chr8_80198129, chr8_83798444, and chr8_80205951, all on chromosome 8, but no other correlation was high, either with the fourth SNP on chromosome 8 (chr8_77736763) or the SNPs on chromosomes 2 and 4 (Table 3, Figure S2). Within the 4 million bp on chromosome 8 where three of the four QTLs were localized, the linkage disequilibrium, calculated after kinship correction, was short, with a sharp drop of *R* ^2^ after 250 bp (Figure S3).

**TABLE 3:** Pearson correlation between allele frequencies of the six quantitative trait loci (QTLs) for anthracnose resistance.

	chr8_83798444	chr8_77736763	chr2_1220775	chr8_80205951	chr4_76542639
chr8_80198129	0.724	−0.191	−0.081ns	−0.554	0.166
chr8_83798444	1.000	−0.158	−0.077ns	−0.503	0.142
chr8_77736763		1.000	−0.037ns	0.080ns	0.043ns
chr2_1220775			1.000	−0.009ns	−0.096ns
chr8_80205951				1.000	−0.146
chr4_76542639					1.000

*Note*: “ns” indicates that the *p*‐value of the correlation is >0.05.

Among the five QTLs mapped into genes annotated on the Chen et al. (2020) *M. sativa* genome, three were in exons (chr8_83798444, chr2_1220775, chr8_80205951) and two in introns (chr8_80198129, chr8_77736763). The sixth QTL was located very closely to a gene at 159 bp downstream only (Table 2). The three SNPs in coding sequences generated an amino acid shift, with unknown effect on protein activity. As four independent QTLs were located at the bottom of chromosome 8 within 6 million bases, this region was investigated in more details. The gene MS.gene36672 (SNP chr8_77736763) had no homolog on either homologous chromosomes, and the genes MS.gene37046 and MS.gene37047 (SNPs chr8_80198129 and chr8_80205951, respectively) had similar homologs on chromosomes 8.1 and 8.3 but no homolog on chromosome 8.4. The gene MS.gene05533 (SNP chr8_83798444) was absent from chromosome 8.1 but present on the other two homologous chromosomes. The alignment of the last 7‐million‐base genomic sequences of the four homologs of chromosome 8 revealed large insertions/deletions on this part of the genome (Figure S3).

### 3.3 Genomic prediction

**FIGURE 5:**
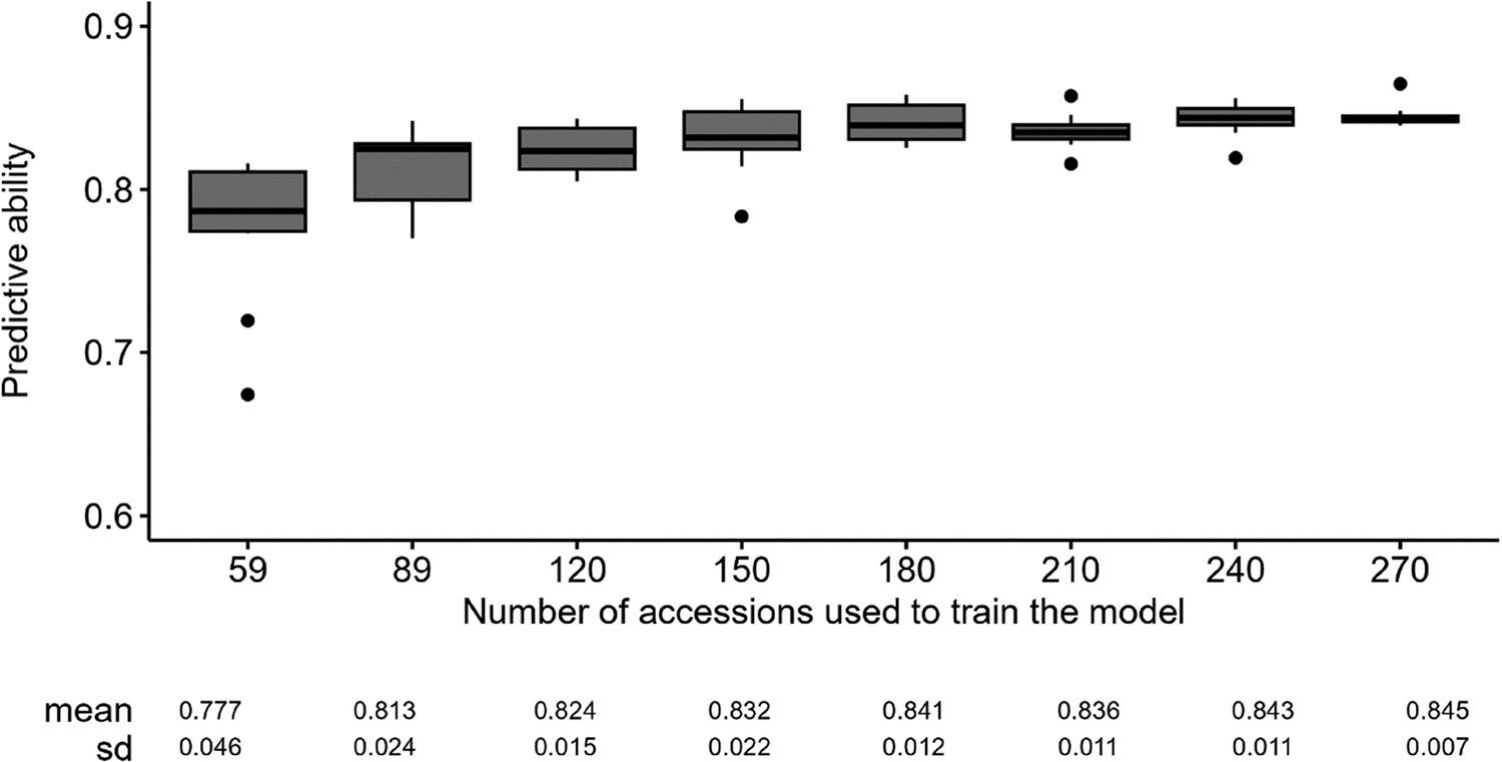
Boxplot for the predictive ability for anthracnose resistance computed on 10 repetitions as a function of the size of the training population. A table showing the average predictive ability for each training population size, along with its standard deviation, is presented in the lower part of the graph.

With a training population composed of 270 accessions, the predictive ability averaged 0.85 over the 10 iterations, ranging from 0.84 to 0.87. The predictive ability was over 0.75 when the training population comprised more than 89 accessions (Figure 5). The increase in the size of the training population increased the predictive ability and decreased its range of variation. The predictive ability tended to stabilize after a training set of 180 accessions.

## 4 DISCUSSION

The experimental design was based on a set of 417 phenotyped populations (percentage of resistant plants), and among them, 380 were genotyped (allele frequencies). Given that alfalfa is a predominantly cross‐pollinating crop with high levels of heterozygosity, the advantage of working directly on populations is noteworthy. Instead of focusing on individual plants, populations can be multiplied and conserved as seeds. They are immediately available without the need to produce progeny. Additionally, they allow to assess anthracnose resistance as the mean of resistant plants within an accession, which is usually done. In addition, this material can be used to evaluate various traits of agronomic interest, including those measured at the plot level, as shown in previous studies (Julier et al., 2022; Pégard et al., 2023). This approach can be enriched by more accessions, provided that the same markers are obtained and the phenotypic tests are connected with common control varieties.

### 4.1 Diversity for anthracnose resistance

The resistance to anthracnose ranged from 0% to 83% in this set of accessions, covering the variation already described among alfalfa varieties. Old varieties and the landraces were generally susceptible, in accordance with results obtained in official French variety tests when anthracnose emerged (Gondran, 1982). The most resistant accessions were either recent varieties or breeding materials, thus highlighting the selection efforts in breeding. Similarly, in red clover (*Trifolium pratense*), landraces and ecotypes were mostly susceptible to anthracnose, and resistant accessions were either registered varieties or breeding materials (Frey et al., 2022). Nonetheless, in alfalfa as well as in red clover, a certain percentage of resistant plants was often present, even in susceptible accessions (Frey et al., 2022; Gondran & Mainer‐Casado, 1973; Irwin et al., 1980). These resistant plants facilitated the breeding of resistant varieties, without the need to introduce exotic germplasm, which would have had a negative effect on agronomically important traits such as yield and persistence.

### 4.2 Genetic control of anthracnose resistance

Two major QTLs on chromosome 8 explained 39.0% and 11.3% of the phenotypic variation, and with four additional QTLs with minor effects, 58.5% of the total variation for anthracnose resistance was explained. All the QTLs were located within or close to genes, and three of them included an LRR domain, a typical pattern observed in resistance genes. Three SNPs were located in exons but did not induce a change in amino acid sequence, and the three other SNPs were in noncoding sequences. However, in this study, as in other GWAS, the chance to reveal the causative mutations is low.

Interestingly, the allele frequencies did not cover the full range of variation from 0 to 1 in the set of accessions. For each SNP, it would be interesting to score anthracnose resistance in breeding materials that would have allele frequency close to 0 or 1. However, for the two major QTLs, and more strikingly for chr8_80198129, the anthracnose resistance reached 75% with an allele frequency around 0.50.

Four QTLs were located on chromosome 8, within a region of 6 million bp on the reference genome where the linkage disequilibrium was short (<250 bp), as usually observed in alfalfa (Herrmann et al., 2010; Pégard et al., 2023). However, the allele frequencies of three of the four QTLs were correlated to each other. Two hypotheses can be proposed: (1) a common selection pressure on these four genes conferred anthracnose resistance, thus generating a correlation among QTLs in a region with a low linkage disequilibrium, and (2) the genome assembly (Chen et al., 2020) is poor in this region, probably due to suboptimal assembly strategies available for autotetraploid organisms a few years ago. This exacerbates the structural variations observed when comparing the sequences of the four homologous chromosomes 8.

Surprisingly, some accessions inhibited a high level of resistance, in spite of having non‐favorable alleles at the two major QTLs for anthracnose resistance, which suggested that, in addition to the QTL identified in this study, other QTLs not detected in this study were probably involved in anthracnose resistance. Several reasons may explain this situation: (1) as the marker density was not extremely high, especially when considering the short LD, some genomic regions were not detected; (2) these cases of unexpected resistance were related to rare alleles that were excluded during the SNP trimming process; and (3) errors in phenotyping and/or genotyping.

The genetic control of resistance to anthracnose pathotype 1 was described to be mainly controlled by a single gene (Elgin & Ostazeski, 1985) in alfalfa. With molecular markers, the role of a major gene was shown in both *M. truncatula* and *M. sativa* (Ameline‐Torregrosa, Cazaux, et al., 2008; Irwin et al., 2006; Mackie et al., 2007; Yang et al., 2007). The region mainly controlling anthracnose resistance was at the bottom of chromosome 8 in our study, with two major QTLs located 3.5 million bases apart and two minor QTLs. Mackie et al. (2007) observed a major QTL at the top of chromosome 8 in an alfalfa mapping population. This region seems to be homologous to the positions of the two major QTLs found in our GWAS study, after considering the inversion of the reference genome and the genetic map (Figure 1). Even if the genome portions apart from the two major QTLs contained some genes with LRR and TIR domains, this chromosomal arm was not recognized as a region that was rich in NBS‐LRR genes (Ameline‐Torregrosa, Wang, et al., 2008). We also found a minor QTL at the bottom of chromosome 4, corresponding to the top of chromosome 4 of *M. truncatula* (Figure 1). In *M. truncatula*, the main QTL region explaining 33%–43% of the variation was located at the top of chromosome 4 (Ameline‐Torregrosa, Cazaux, et al., 2008; Yang et al., 2007) and controlled resistance against pathotypes 1, 2, and C86‐2, the latter being also used in the present study. At the top of chromosome 4, a QTL explaining 7%–8% of the variation was found in alfalfa (Irwin et al., 2006; Mackie et al., 2007), possibly in the same region. Our QTL was located about 10 million bp from the two major QTLs *RCT1* and *Ct1* in *M. truncatula*. In addition, we found a minor QTL on chromosome 2, not described in the literature. A simple genetic inheritance, conferred by a major genome region and modified by minor QTL, was thus evidenced, in accordance with previous studies. However, the genetic control seemed to be different between alfalfa and *M. truncatula*. A simple genetic control was hypothesized for anthracnose resistance in red clover too (Jacob et al., 2015). A GWAS conducted in red clover revealed a major QTL at the top of chromosome 1 on the reference red clover genome of De Vega et al. (2015), explaining 16.8% of the variation, in addition to QTL of less importance (Frey et al., 2022). This QTL was located in a gene that was annotated as a 3‐oxoacyl‐(acyl carrier) synthase II (De Vega et al., 2015). Given these results, it seems that the control of anthracnose resistance in red clover is possibly different than that in alfalfa.

### 4.3 Genomic prediction

The predictive ability of our genomic selection reached 0.85, which is likely the highest achieved in alfalfa to date, even higher than obtained for autumn dormancy on the same material (Pégard et al., 2023). Similar to this earlier publication, with 89 accessions and beyond, the mean predictive ability plateaued, but the range of variation was reduced by including a larger number of accessions in the training populations. These exceptional results might be explained by the presence of six QTLs, which account for nearly 60% of the phenotypic variation. This study is the first to demonstrate genomic prediction for anthracnose resistance in alfalfa, complementing numerous studies that predict the feasibility of genomic prediction in this species.

In this study, we chose to test the performance of GBLUP exclusively because with the same population, we found that Lasso and GBLUP performed similarly for autumn dormancy, even when major QTLs were detected (Pégard et al., 2023). This might be explained by an infinitesimal genetic architecture, or the presence of QTL can significantly influence predictive ability, as seen in the study where six QTLs explained 60% of the phenotypic variation, leading to high predictive ability even with a small number of accessions in the training set.

In addition to the effect of the method, previous research indicated that predictive accuracies can vary significantly depending on the populations and the traits, with forage quality showing the narrowest range (0.30–0.40) and biomass yield showing the widest range (0.20–0.80) (Annicchiarico et al., 2015, 2021, 2022; X.‐Q. Jiang et al., 2022; Murad Leite Andrade et al., 2022; Pégard et al., 2023). Overall, these results contribute to the growing body of research demonstrating the feasibility and potential of genomic prediction in alfalfa but highlight the need for continued exploration and optimization of genomic prediction models.

### 4.4 Consequences for breeding

For future breeding efforts in alfalfa, the use of molecular markers could help to increase anthracnose resistance. We propose two strategies, which can be combined. First, from the two major QTLs on chromosome 8, an analysis of the candidate gene polymorphism could be conducted, and the correlation between such polymorphism and phenotypic variation could help to identify causal mutation in the genes. With the development of high‐throughput markers, such as KASP markers, polycrosses composed of plants carrying three to four doses of the allele conferring the resistance would produce highly resistant progeny. Second, a genomic prediction could be conducted on relevant breeding pools, and the plants predicted to be the most resistant ones could be intercrossed. Both strategies could be combined to select plants carrying the anthracnose resistance alleles at the two major QTLs and plants that have the highest genomic prediction values. In the case of anthracnose resistance that is relatively easy to score, marker‐assisted selection may not be economically feasible. However, given the complex inheritance due to tetraploidy in alfalfa, molecular markers could be the only way to fix resistance alleles in breeding populations. Moreover, the number of studies showing promising QTL or genomic prediction results increases, and genomic selection allows to improve multiple traits simultaneously, thus speeding up breeding.

Results obtained with GWAS and genomic prediction are always associated with the set of accessions. Considering that susceptible accessions were over‐represented in our set, and the most resistant accessions mostly originated from the United States, it would be valuable to introduce more resistant accessions from diverse origins, if available. If the new accessions are phenotyped with the same protocol as the one used here and genotyped with the same marker set, it would be possible to check if the composition of the set affected the QTL detection and the genomic prediction in our study.

Breeding alfalfa for disease resistance is of major importance to increase protein‐rich forage production and stand persistency, thus contributing to lower agricultural inputs. So far, the varieties that were scored as anthracnose resistant in the past have maintained their resistance to the same pathotypes, as if the resistance genes had no significant effect on the pathogen evolution. In the future, if by using molecular markers alfalfa varieties containing 100% resistant plants would be created, we could expect the evolution of the *C. trifolii* pathogen toward pathotypes that overcome this resistance, as often described in crops grown as homogeneous varieties. This risk was mentioned in the past (Guy, 1976; Raynal, 1977). A solution could be to create alfalfa varieties with a small percentage of susceptible plants while maintaining the low selection pressure on the pathogen.

## AUTHOR CONTRIBUTIONS

**Marie Pégard**: Data curation; formal analysis; investigation; methodology; validation; writing—original draft; writing—review and editing. **Camille Gréard**: Data curation; formal analysis; investigation; methodology; writing—original draft; writing—review and editing. **Marius Grelier**: Data curation; formal analysis; visualization; writing—review and editing. **Marie‐Christine Gras**: Conceptualization; investigation; methodology; writing—review and editing. **Laure Saint‐Pierre**: Investigation; methodology; validation; writing—review and editing. **Bernard Tharel**: Conceptualization; investigation; methodology; writing—review and editing. **Philippe Barre**: Conceptualization; methodology; writing—review and editing. **Bernadette Julier**: Conceptualization; funding acquisition; methodology; project administration; supervision; writing—original draft; writing—review and editing.

## CONFLICT OF INTEREST STATEMENT

The authors declare no conflicts of interest.

## Supporting information



Supplemental Figure S1. QQplot for the QTL detection, showing six significant SNP.
Supplemental Figure S2. Correlation between allele frequencies of chr8_83798444 (y‐axis) and chr8_80205951 and between chr8_80198129 and chr8_80205951. The points, representing the accessions, are colored depending on their percentage of resistance, from red for a low percentage of resistance to green for a high percentage of resistance.
Supplemental Figure S3. Linkage disequilibrium on chromosome 8 within a distance of 4 million base pairs, in the region around SNP chr8_80198129.
Supplemental Figure S4. Alignment of the homologous chromosomes 8 in the region of the QTL, with chr8.2, used as the reference for GBS mapping, compared to the other three chromosomes. Identity legend: 0‐0.25: yellow, 0.25‐0.5: orange, 0.5‐0.75 light green, 0.75‐1: dark green.

Supplemental Table S1: list of alfalfa accessions with their country of origin, status, registration year when available, and adjusted resistance to anthracnose, pathotype C86‐2.

## Data Availability

The genotyping data are available at https://doi.org/10.57745/L0FLJD. The phenotyping data are available in the Table S1.
