# Supplementary material for: QTL detection and genomic prediction for resistance to anthracnose in alfalfa (Medicago sativa)
Source: Plant Genome. 2025 Aug 6;18(3):e70085. doi: 10.1002/tpg2.70085 (PMC12326290; doi:10.1002/tpg2.70085)

**Supplemental Figure S1. QQplot for the QTL detection, showing 6 significant SNP.**


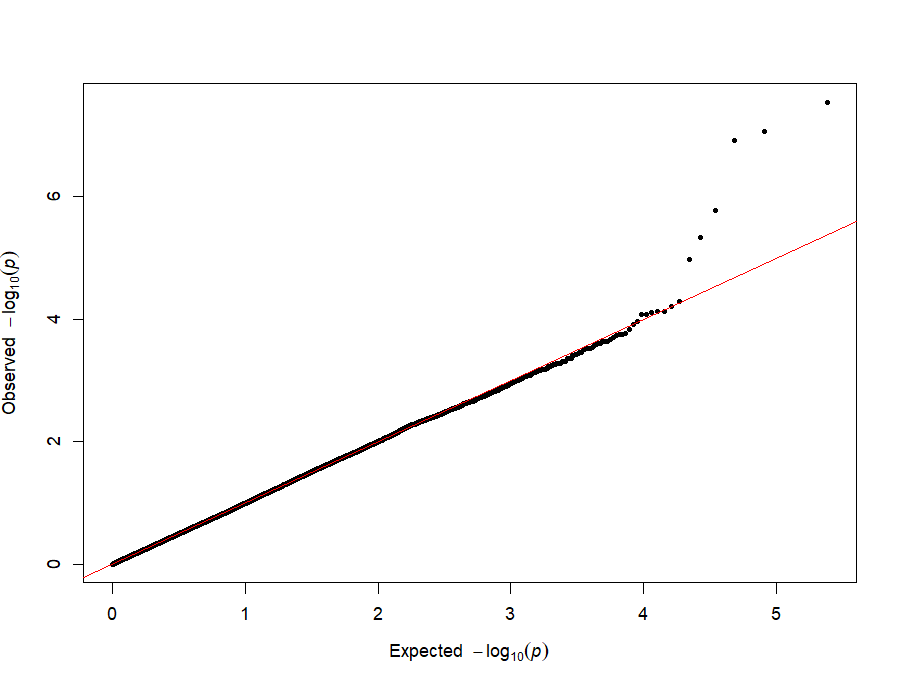


**Supplemental Figure S2. Correlation between allele frequencies of chr8_83798444 (x-axis) and chr8_80205951 (y-axis) and between chr8_80198129 (x-axis) and chr8_80205951 (y-axis). The points, representing the accessions, are colored depending on their percentage of resistance, from red for a low percentage of resistance to green for a high percentage of resistance**. The blue line represents the regression between the percentage of resistance of these two QTL and the grey part the confidence interval of this regression, the equation and R^2^ of that regression is represented on each graph**.**


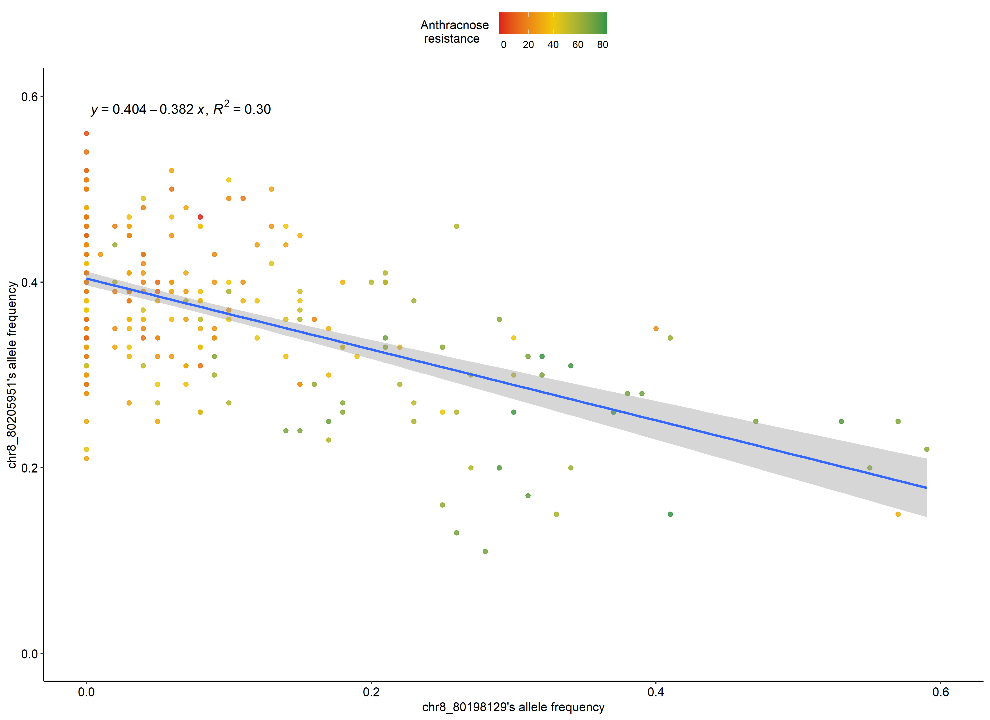

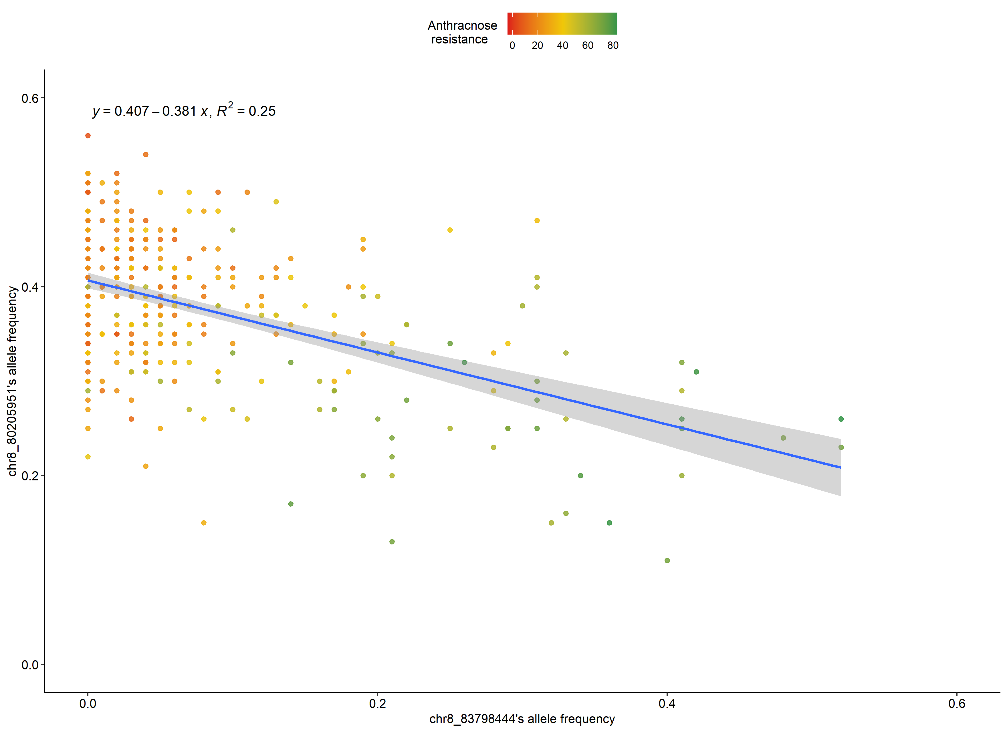


**Supplemental Figure S3. Linkage disequilibrium on the chromosome 8 within a distance of 4 million base pairs, in the region around SNP chr8_80198129.**


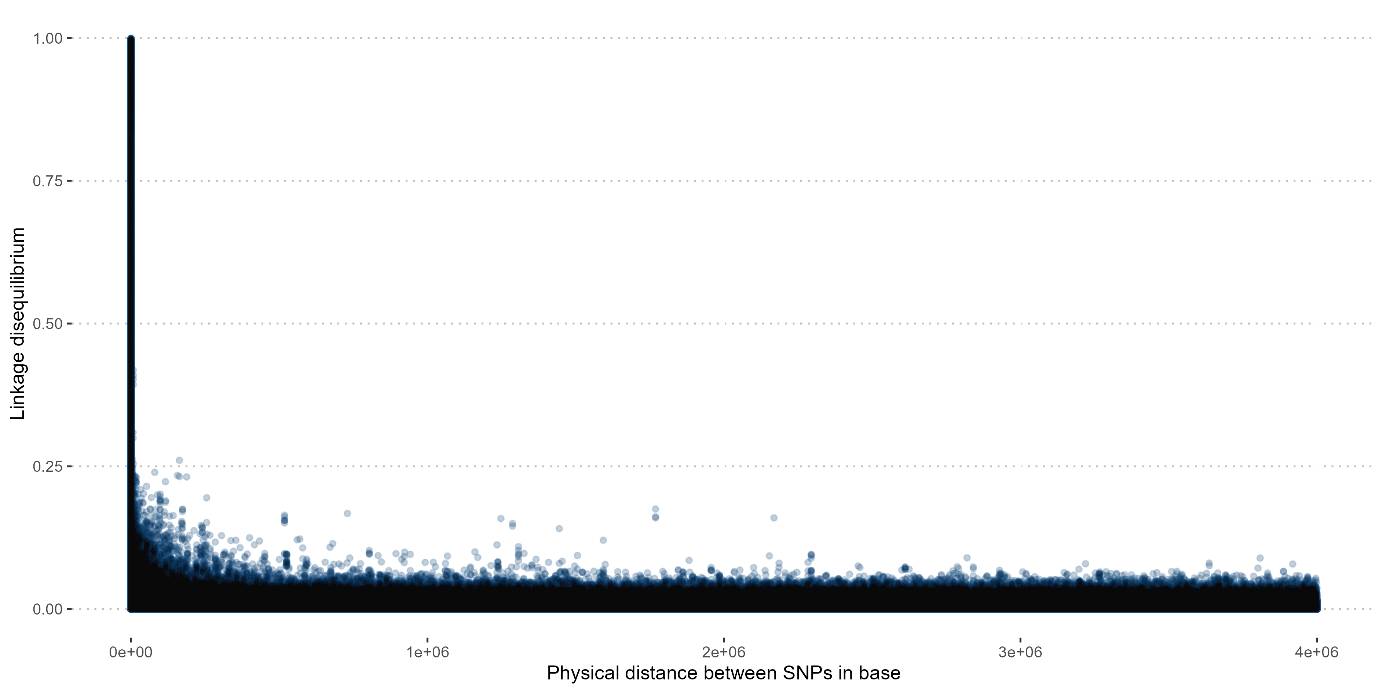


**Supplemental Figure S4. Alignment of the homologous chromosomes 8 in the region of the QTL, with chr8.2, used as the reference for GBS mapping, compared to the other three chromosomes. Identity legend: 0-0.25: yellow, 0.25-0.5: orange, 0.5-0.75 light green, 0.75-1: dark green.**


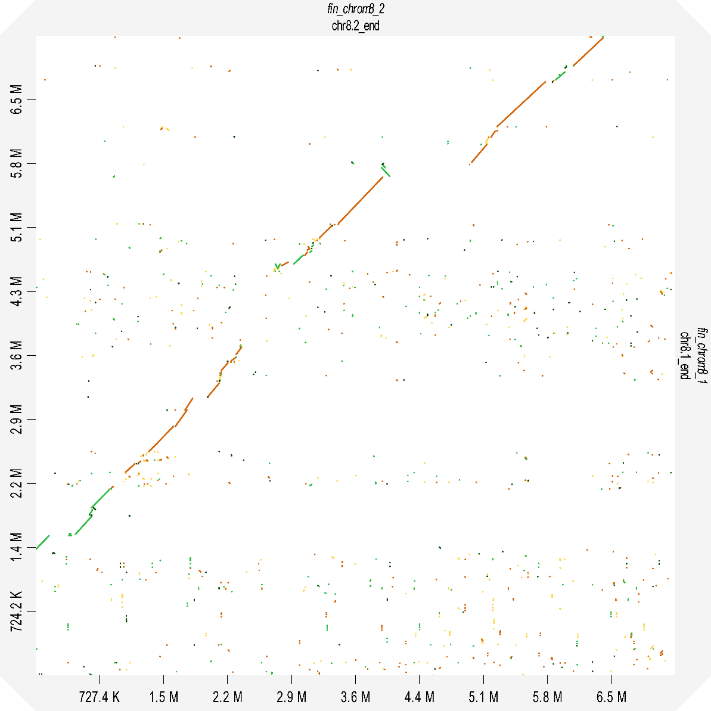

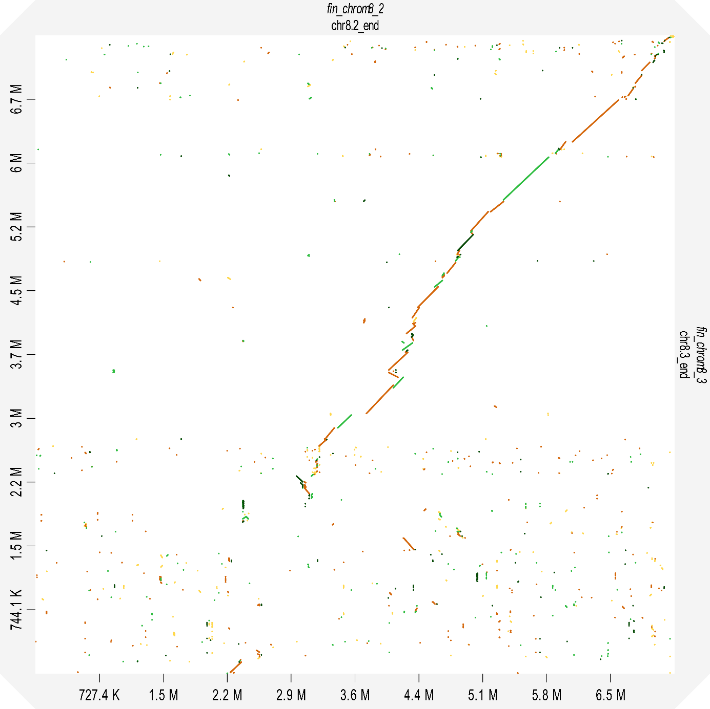

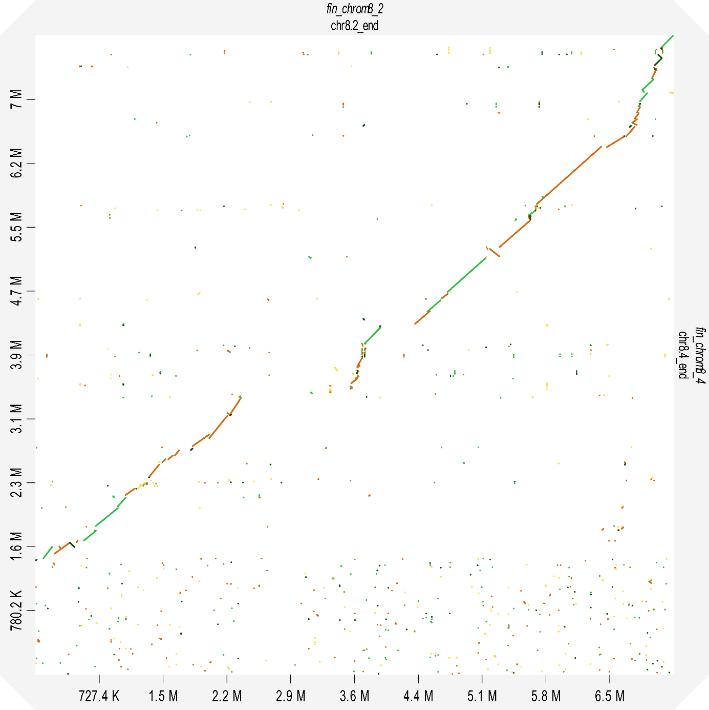

Supplement: Supplementary file 1 — Supplemental Figure S1. QQplot for the QTL detection, showing six significant SNP. Supplemental Figure S2. Correlation between allele frequencies of chr8_83798444 (y‐axis) and chr8_80205951 and between chr8_80198129 and chr8_80205951. The points, representing the accessions, are colored depending on their percentage of resistance, from red for a low percentage of resistance to green for a high percentage of resistance. Supplemental Figure S3. Linkage disequilibrium on chromosome 8 within a distance of 4 million base pairs, in the region around SNP chr8_80198129. Supplemental Figure S4. Alignment of the homologous chromosomes 8 in the region of the QTL, with chr8.2, used as the reference for GBS mapping, compared to the other three chromosomes. Identity legend: 0‐0.25: yellow, 0.25‐0.5: orange, 0.5‐0.75 light green, 0.75‐1: dark green. [file TPG2-18-e70085-s001.docx]
